# Inhibitory effect of the novel tyrosine kinase inhibitor DCC-2036 on triple-negative breast cancer stem cells through AXL-KLF5 positive feedback loop

**DOI:** 10.1038/s41419-022-05185-x

**Published:** 2022-08-30

**Authors:** Yingying Shen, Qingyun Zhu, Maoyu Xiao, Liyang Yin, Wenjie Feng, Jianbo Feng, Jun He, Pei Li, Xiguang Chen, Wenjun Ding, Jing Zhong, Zhaolin Zeng, Zhuoye Xie, Jianghua Liu, Xuyu Zu

**Affiliations:** 1 The First Affiliated Hospital, Cancer Research Institute, Hengyang Medical School, University of South China, Hengyang, Hunan 421001 PR China; 2 The Nanhua Affiliated Hospital, Department of Spine Surgery, Hengyang Medical School, University of South China, Hengyang, Hunan 421001 China; 3 The First Affiliated Hospital, Department of Metabolism and Endocrinology, Hengyang Medical School, University of South China, Hengyang, 421001 China; 4 Institute of Biomaterials and Biomedical Engineering, University of Toronto, Toronto, M5S 3G9 Canada

**Keywords:** Breast cancer, Cancer stem cells

## Abstract

Triple-negative breast cancer (TNBC), an aggressive histological subtype of breast cancer, exhibits a high risk of early recurrence rate and a poor prognosis, and it is primarily associated with the abundance of cancer stem cells (CSCs). At present, the strategies for effectively eradicating or inhibiting TNBC CSCs are still limited, which makes the development of novel drugs with anti-CSCs function be of great value for the treatment of TNBC, especially the refractory TNBC. In this study, we found that the small-molecule tyrosine kinase inhibitor DCC-2036 suppressed TNBC stem cells by inhibiting the tyrosine kinase AXL and the transcription factor KLF5. DCC-2036 downregulated the expression of KLF5 by decreasing the protein stability of KLF5 via the AXL-Akt-GSK3β signal axis, and in turn, the downregulation of KLF5 further reduced the expression of *AXL* via binding to its promotor (−171 to −162 bp). In addition, p-AXL/AXL levels were positively correlated with KLF5 expression in human TNBC specimens. These findings indicated that DCC-2036 is able to suppress the CSCs in TNBC by targeting the AXL-KLF5 positive feedback loop. Moreover, our findings indicated that DCC-2036 increased the sensitivity of TNBC chemotherapy. Therefore, this study proposes a potential drug candidate and several targets for the treatment of refractory TNBC.

## Introduction

Breast cancer has surpassed lung cancer to become the tumor with the highest incidence in the world. It had about 2.3 million new cases in 2020, taking up 11.7% of all new cancer cases in the world [1]. Therefore, the exploration of the pathogenesis and clinical treatment of breast cancer has become a hot spot in the field of cancer research. Triple-negative breast cancer (TNBC) is an aggressive subtype of breast cancer which is negative for estrogen receptor (ER), progesterone receptor (PR), and human epidermal growth factor receptor 2 (HER2), which nullifies existing endocrine therapies and targeted therapies. Around 24% of breast cancer patients were diagnosed with TNBC [2, 3], which correlates with a higher rate of relapse and chemoresistance, as well as shorter overall survival. Additionally, TNBC constitutes 90% of breast cancer-related mortality [4]. In this case, discovering and researching effective therapies and drugs for TNBC is an urgent need as well as a major challenge in the field of breast cancer treatment.

Accumulating evidences have shown that cancer stem cells (CSCs) play an essential role in breast cancer metastasis, recurrence, and chemoresistance [5–7]. Previous studies have demonstrated that salinomycin, which selectively suppressed breast CSCs by inducing CSCs differentiation, was effective in inhibiting breast cancer [8]. Moreover, TNBC has a higher percentage of CSCs compared to the other subtypes of breast cancer [9, 10]. Thereby, exploring new drugs with the function of eradicating or inhibiting CSCs as well as the related targets and molecular mechanisms have important academic and clinical application value.

The third-generation tyrosine kinase inhibitor DCC-2036 (Rebastinib), designed as an inhibitor of ABL1 switch-control, is powerful in chronic myeloid leukemia (CML). In addition to ABL1, DCC-2036 can also target FLT3, TIE2, SRC, PDGFRα, FYN, AXL, MET, and other tyrosine kinases [11]. In our previous study, DCC-2036 has a potent anti-TNBC effect [12]. Besides, DCC-2036 could induce apoptosis and significantly reduces migration and invasion of TNBC cell lines in vitro. More importantly, DCC-2036 showed pronounced antitumor activity in the xenograft NSG model and the TNBC PDX model without significant toxicity [12]. Consequently, we reasonably speculate that it may also inhibit TNBC stem cells.

Studies have found that KLF5 is specifically and highly expressed in TNBC cell lines. KLF5 promoted TNBC cell proliferation, survival, migration, invasion, and stemness [13–15]. Moreover, KLF5 is an unfavorable indicator of survival for breast cancer patients [16]. Downregulating KLF5 could be an avenue that can be used to inhibit TNBC stem cells and Mifepristone, an FDA-approved drug, was found to have such an effect [15]. Therefore, whether DCC-2036 could inhibit TNBC CSCs by downregulating KLF5 expression was investigated. In addition, our previous research revealed that DCC-2036 mainly exerted its anti-TNBC effect by inhibiting the activity and expression of the tyrosine kinase AXL [12]. AXL is highly expressed in TNBC cells and can promote the stemness of breast cancer [17, 18], but the mechanism is still not clear enough and needs further exploration.

In the present study, we found that tyrosine kinase inhibitor DCC-2036 could suppress TNBC stem cells by inhibiting the positive feedback loop between AXL and KLF5. We also observed a positive correlation between p-AXL/AXL protein levels and KLF5 protein expression in human TNBC specimens. Taken together, these findings demonstrate that DCC-2036 can significantly inhibit CSCs in TNBC by targeting the AXL-KLF5 positive feedback loop. Therefore, this study can provide a potential drug candidate and targets for the treatment of refractory TNBC.

## Materials and methods

### Cell culture

The TNBC cell lines MDA-MB-231, HS-578T, and 4T1 were purchased from the Cell Bank at the Chinese Academy of Sciences (Shanghai, China). All cells were cultured in Dulbecco’s modified Eagle’s medium (DMEM) supplemented with 10% heat-inactivated fetal bovine serum (Biological Industries, Northern Kibbutz Beit Haemek, Israel) and 1% penicillin/streptomycin at 37 °C with 5% CO_2_. The cell lines were characterized by the Genetic Testing Biotechnology Corporation (Suzhou, Jiangsu, China) using short tandem repeat (STR) markers and no mycoplasma contamination.

### Chemicals and antibodies

DCC-2036 (Rebastinib) was purchased from Selleck (Houston, TX). The compound was dissolved in DMSO at a stock concentration of 20 mM and stored at −20 °C. DCC-2036 (0 μM) means DMSO treatment with equivalent volume. Recombinant Human GAS6 (C-Fc) #C12W was purchased from Novoprotein (Suzhou, Jiangsu, China). Recombinant Mouse GAS6 Protein (His Tag) #58026-M08H was purchased from Sino Biological (Shanghai, China). Cycloheximide and MG132 were purchased from Sigma-Aldrich (St Louis, MO, USA). The following antibodies were used for immunoblotting: phospho-AXL (Y702) #5724, phospho-AKT (S473) #4060, AKT #4691,phospho-GSK3β (S9) #5558, GSK3β #12456, β-actin #4970, purchased from Cell Signaling Technology (Beverley, MA); ALDH1A1 #15910-1-AP, OCT4 #11263-1-AP, KLF4 #11880-1-AP, SOX2 #11064-1-AP, KLF5 #21017-1-AP, NANOG #14295-1-AP, AXL #13196-1-AP, MET #25869-1-AP, FGF-BP1 #25006-1-AP purchased from Proteintech (Wuhan, Hubei, China); NF-κB (P65) #ab16502, purchased from Abcam; FGF-BP1 #AF1593 purchased from R&D; phospho-KLF5 (S303) #AF7042, phospho-AXL (Tyr702) # AF8523 purchased from Affinity (Changzhou, Jiangsu, China). APC anti-mouse CD24 #138506 purchased from Biolegend (San Diego, CA, USA), PE Rat anti-mouse CD44 #553134, PE Mouse Anti-Human CD24 #555428, APC Mouse Anti-Human CD44 #559942 purchased from BD Pharmingen (Franklin Lakes, New Jersey, USA).

### Real-time RT-PCR

Total RNA was extracted from cells and tissues using TRIzol reagent (Invitrogen; Thermo Fisher Scientific, Waltham, MA) and reverse transcribed with the RevertAid First Strand cDNA Synthesis Kit (Thermo Fisher Scientific) as the manufacturer’s protocol instructed. Primers used are listed below: AXL forward, 5’-GACATAGGGCTAAGGCAAGAGG-3’; AXL reverse, 5′-CGAGAAGGCAGGAGTTGAAGG-3′; KLF5 forward, 5′-ACACCAGACCGCAGCTCCA-3′; KLF5 reverse, 5′-TCCATTGCTGCTGTCTGATTTGTAG-3′; Nanog forward, 5′-TTTGTGGGCCTGAAGAAAACT-3′; Nanog reverse, 5′-AGGGCTGTCCTGAATAAGCAG-3′; GAPDH forward, 5′-AGCCTCAAGATCATCAGCAATGCC-3′; and GAPDH reverse, 5′-TGTGGTCATGAGTCCTTCCACGAT-3′. Quantitative measurement of target gene mRNA levels was performed using the ABI Prism 7500 Sequence Detection System (Applied Biosystems; Thermo Fisher Scientific). The data were analyzed using the 2-ΔΔCq method.

### Transfection

KLF5 plasmids were produced by Genechem (Shanghai, China) and transfected into cells using Lipofectamine 3000 (Invitrogen; Thermo Fisher Scientific) according to the manufacturer’s protocol. Twenty-four hours after transfection, cells were exposed to DCC-2036 for 48 h. The AXL siRNA (5′-GCCUGACGAAAUCCUCUAUTT-3′), MET siRNA (5′-CTCATTTGGATAGGCTTGTAA-3′), P65 siRNA(5′-GCCAUCUACUGACAGUAAATT-3′), KLF5 siRNA(5′-CGATTACCCTGGTTGCACA-3′), and negative control siRNA (5′-UUCUCCGAACGUGUCACGUTT-3′) were synthesized by GenePharma (Shanghai, China). siRNAs were transfected into cells using Lipofectamine RNAiMAX (Invitrogen; Thermo Fisher Scientific) following the manufacturer’s protocol.

### Flow cytometry

The ALDEFLUOR Assay Kit (#01700; Stemcell Technologies, Vancouver, BC, Canada) was used for the detection of the ALDH+ cells by flow cytometry. About 1 × 10^6^ cells were resuspended in 1 ml assay buffer. Then, the sample was added to an activated Aldecount Reagent tube. Following that, 0.5 ml cells were immediately put into a new tube containing 5 μl DEAB buffer. All samples were incubated for 45 min at 37 °C. All samples were incubated for 45 min and centrifuged at 250 × *g* for 5 min. The cells were then resuspended in 0.5 ml assay buffer and analyzed immediately by flow cytometry.

CD44+/CD24− is also characteristic of breast CSCs. TNBC cells were treated with indicated concentrations of DCC-2036 for 48 h and then trypsinized, suspended into single-cell mixtures, and washed with PBS. Cells were incubated on ice for 30 min with antibodies specific to human or mouse cell surface markers, including CD44 and CD24 (1:50). After washing, staining was assessed in a BD FACSArial II flow cytometer, followed by analysis using BD FACSDiva software.

### Mammosphere culture

Mammosphere Culture Kit (#05620; Stemcell Technologies) was used for the mammosphere assay. To induce sphere formation, TNBC cells were digested into single cells and were plated into 24-well ultra-low attachment plates (#3473, Corning) at a density of 5000 cells per well. The cells were cultured containing 500 μl of complete MammoCult™ medium (#05620; Stemcell Technologies). After 10–14 days, the number and size of mammospheres were assessed.

### Western blotting

For immunoblotting, cells were harvested, washed with ice-cold PBS buffer, lysed in RIPA buffer (Beyotime, #P0013B, China), and centrifuged at 13,000 rpm at 4 °C for 10 min. Protein lysates were fractionated by SDS-polyacrylamide gel electrophoresis, transferred onto PVDF membranes (Merck Millipore, #IPFL00010, Germany), and then incubated with indicated primary antibodies, washed, and probed with HRP-conjugated secondary antibodies. ECL Detection System (Merck Millipore, #WBKLS0500, Germany) was used for signal detection.

### Immunoprecipitation and immunoblotting

For immunoprecipitation, cells were harvested, washed with ice-cold PBS buffer, and lysed in IP buffer (#P0013, Beyotime) for 30 min, followed by centrifugation for 10 min at 13,000 rpm. Cell lysates were incubated with indicated antibodies at 4 °C overnight. About 40 μl protein A/G Magnetic Beads (#B23202, Bimake) after washing three times with IP buffer were then added to the reaction mixtures and incubated for 4 h at 4 °C. After magnetic separation, the magnetic beads were washed four times with IP buffer and boiled for 10 min with addition of 2× SDS loading buffer, immunoprecipitated proteins were analyzed by SDS-PAGE.

### A limiting dilution assay for tumorigenesis

MDA-MB-231 cells (5 × 10^3^–2 × 10^5^) were suspended in DMEM and Matrigel (#354234, Corning, BD Biocoat) at a 1:1 ratio and were injected into the fat pads of 4–6 weeks old female BALB/C-nude mice from the Hunan SJA Laboratory Animal Co. Ltd (Changsha, Hunan, China). 4T1 cells (2 × 10^3^−2 × 10^5^) were suspended in DMEM and injected into the fat pad of 6–7 weeks old female BALB/C mice. After 7 days, the site of implantation was monitored for tumor growth and tumor size was measured every 2 or 3 days. Mice were randomly assigned to control and experimental groups without investigator blinding. This animal experimentation was approved by the animal ethics committee of the University of South China.

### Immunohistochemical staining

The clinical tissue array was obtained from Xi’an Alena Biotech Company (US Biomax, #BR487c). The phospho-AXL, AXL, and KLF5 staining was performed according to the protocol described in our previous study [12]. The following antibodies were used for immunohistochemical staining at the indicated dilution: human and murine KLF5 (Proteintech; #21017-1-AP; 1:100), human and murine p-AXL (R&D Systems; #AF2228; 1:30), human AXL (R&D Systems; #AF154, 1:40), murine AXL (R&D Systems; #AF854, 1:40).

### ChIP qPCR

A ChIP kit (Abcam, #ab500) was used according to the manufacturer’s instructions. The KLF5 CHIP antibody was purchased from Invitrogen (#701885). The following qPCR primers were used: AXL-1 forward, 5′-CCTTGTCCGAGGAGCCGAGA-3′; AXL-1 reverse, 5′-CTGGGCTCTGTGTCTGGTAAACA-3′; AXL-2 forward, 5′- CTCACTGGCTCAGGACAGG-3’; AXL-2 reverse, 5’-AGGATCAGCTCTTTCTTAAAGGG-3’; AXL-3 forward, 5’-CTTGAGTTAACCCCTGATTGTCC-3′; AXL-3 reverse, 5′- TGCCTCCTTCCCTCACTCCC-3′. The results were shown as relative enrichment compared with IgG [19]. For negative control, the following AXL qPCR primers were used: Forward, 5′-ACCTCGTGATCCACTCGC-3′; Reverse, 5′-CCTCAAACTCCTGGGCTTAT-3′.

### Luciferase assay

The AXL-WT promoter and AXL-Mutant promoter were constructed by General Biosystems (Anhui) Co., Ltd. A luciferase assay kit (Promega) was used to measure the reporter activity according to the manufacturer’s instructions. Luciferase activity was normalized by using a Renilla luciferase internal control.

### Statistical analysis

All experiments that required a statistical analysis were performed at least three times. The experimental data were expressed as mean ± standard deviation (Mean ± SD). The statistical analyses were performed using GraphPad Prism 8.0 software (GraphPad Software). Statistical comparisons between groups were analyzed for significance by two-tailed Student’s *t*-test and one-way ANOVA with Tukey’s multiple comparison test. *P* < 0.05 is considered to be a statistically significant difference. Inclusion/exclusion criteria were all pre-established and no samples or animals were excluded from the analysis.

## Results

### DCC-2036 inhibits TNBC stem cells

**Fig. 1:**
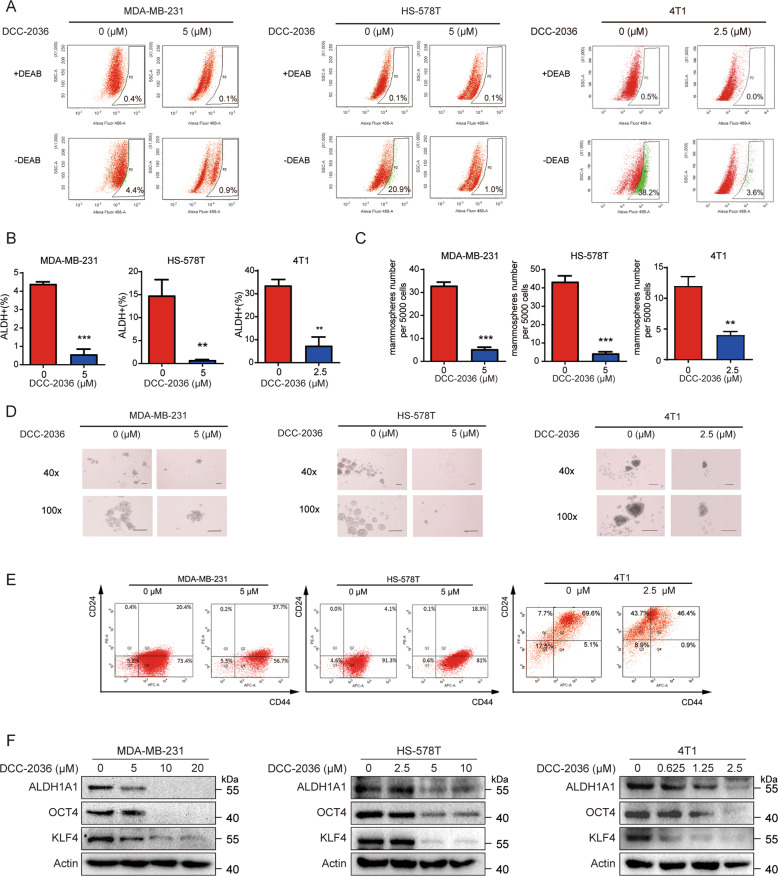
Inhibitory effect of DCC-2036 on TNBC stem cells. **A** According to the ALDEFLUOR assay, DCC-2036 suppressed cancer stem cells (CSCs) obviously in the TNBC cell lines MDA-MB-231, HS-578T, and 4T1. After DCC-2036 treatment for 48 h, cells were incubated with activated ALDEFLUOR substrate followed by flow cytometry to measure the percentage of ALDH+ cells. DEAB treatment functions as a negative control for ALDH+ gating. Incubation of cells with ALDEFLUOR substrate in the absence of DEAB induces a change in fluorescence that defines the ALDH+ population. DCC-2036 (0 μM) means DMSO treatment with equivalent volume. **B** Former results are shown in bar charts (*n* = 3, independent experiments). The statistical significance was determined by Student’s *t*-test. **P < 0.01, ***P < 0.001. **C**, **D** The suppressive effect of DCC-2036 on TNBC stem cells according to the mammosphere formation assay. MDA-MB-231, HS-578T, and 4T1 cells were incubated with DCC-2036 for 48 h before they were plated onto 24-well ultra-low attachment plates (5000 cells each well). The number of mammospheres with diameters >60 μm were counted after 7 days. **D** Representative images of mammospheres in a condition of treating with DCC-2036. Scale bars, 100 μm. **C** The results are shown as bar charts (*n* = 3, independent experiments). The statistical significance was determined by Student’s *t*-test. ***P < 0.001. **E** Flow cytometry analysis of the CD44^+^CD24^−^ population in MDA-MB-231, HS-578T, and 4T1 cells after DCC-2036 treatment. **F** MDA-MB-231 cells, HS-578T cells, and 4T1 cells were treated with DCC-2036 with indicated concentration for 48 h. The protein levels of CSCs-associated markers were examined using Western blotting.

Positive aldehyde dehydrogenase (ALDH+) is a characteristic of cancer stem cells (CSCs) [20]. To investigate whether DCC-2036 reduced the percentage of TNBC stem cells, we used flow cytometry to evaluate the expression of ALDH. It was shown that DCC-2036 could reduce the proportion of ALDH+ cell populations in MDA-MB-231 cells, HS-578T cells, and 4T1 cells (Fig. 1A, B), which are TNBC cell lines. Besides, the phenotypic CD44^+^/CD24^−^ subpopulation of breast cancer cells are also termed as BCSCs [6]. It was also found that DCC-2036 reduced the percentage of CD44^+^/CD24^−^ subpopulation in TNBC cells (Fig. 1E). Then, we evaluated the enrichment of CSCs in the TNBC cell lines through mammosphere formation assay and found that DCC-2036 also significantly reduced the number and size of mammospheres formed in MDA-MB-231, HS-578T, and 4T1 cells (Fig. 1C, D). Those results indicated that DCC-2036 could repress the formation of CSCs and downregulated the protein expression of the typical CSC-characterizing markers in MDA-MB-231, HS-578T, and 4T1 cells (Fig. 1F).

### DCC-2036 inhibits TNBC stem cells partially by reducing the stability of KLF5 protein via the ubiquitin-proteasome pathway

**Fig. 2:**
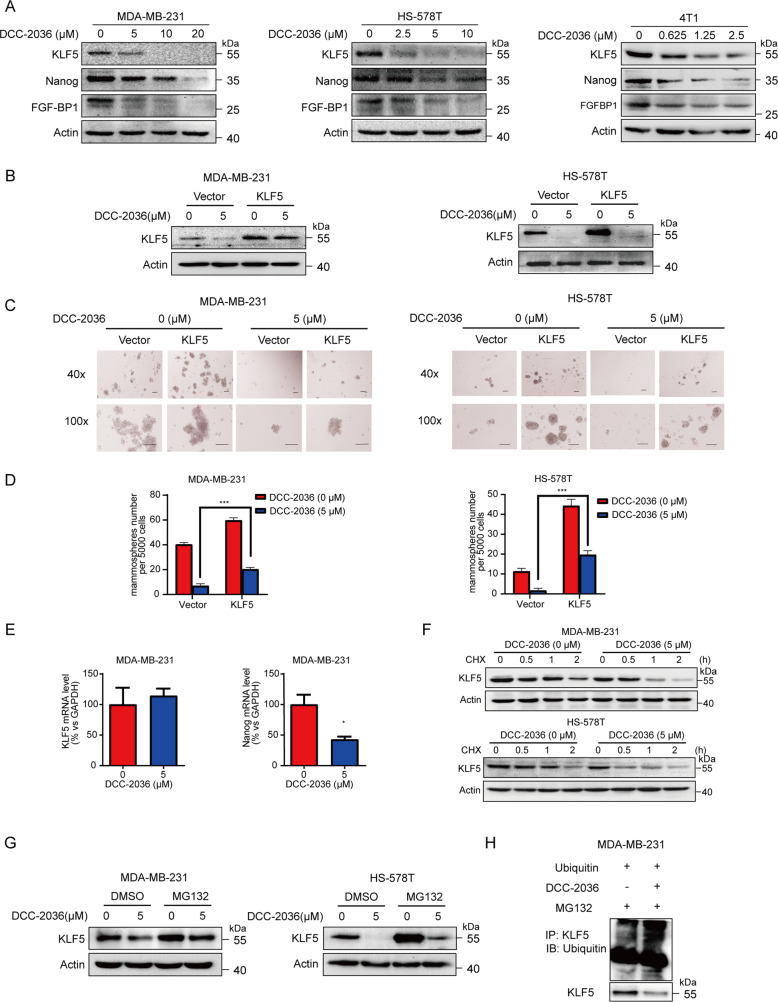
DCC-2036 restrains TNBC stem cells partially by undermining the stability of the KLF5 protein. **A** DCC-2036 inhibited the expression of KLF5, as well as its downstream targeted genes Nanog and FGF-BP1 in TNBC cells. The cells were incubated with DCC-2036 at indicated concentrations for 48 h, and the protein expression was examined by Western blotting. **B** KLF5 or vector plasmids were transfected into MDA-MB-231 and HS-578T cells for 24 h. The cells were then treated with either DCC-2036 (5 μM) or DMSO for 48 h, and the expression of KLF5 was detected by WB. **C**, **D** Ectopic expression of KLF5 partially but significantly rescued the mammosphere formation reduction induced by DCC-2036. MDA-MB-231 cells and HS-578T cells were transfected with KLF5 or vector plasmid, followed by DCC-2036 (0 or 5 μM) treatment for 48 h before they were plated onto 24-well ultra-low attachment plates (5000 cells each well). The number of mammospheres were counted 7 days after seeding and shown in bar charts. Scale bars, 100 μm. **D** The histogram represents the mean value of three experiments. The statistical significance was determined by Student’s *t-t*est. ***P < 0.001. **E** DCC-2036 did not reduce the mRNA level of KLF5 in MDA-MB-231 cells but downregulated the mRNA level of Nanog. The cells were treated with DCC-2036 (0 or 5 μM) for 48 h and the mRNA levels were measured by q RT-PCR. The statistical significance was determined by Student’s *t*-test, ***P < 0.001. **F** WB showed that DCC-2036 could promote the degradation of the KLF5 protein in MDA-MB-231 cells and HS-578T cells, which were treated with DCC-2036 (5 μM) or DCC-2036 (0 μM) for 12 h, followed by cycloheximide (CHX, 100 μg/ml) for 0.5, 1, or 2 h. **G** The proteasome inhibitor MG132 mitigated the DCC-2036-induced KLF5 decrease in MDA-MB-231 cells and HS-578T cells. The cells were treated with DCC-2036 (5 μM) or DCC-2036 (0 μM) for 44 h, followed by MG132 (20 μM) for 4 h and then for the Western blot assay. **H** Treated with 10 μM DCC-2036 for 24 h, KLF5 ubiquitination was assessed by an anti-KLF5 antibody in the presence of MG132 after ubiquitin were transfected into MDA-MB-231 cells.

KLF5 is an essential transcription factor which is associated with TNBC CSCs [15]. Its downstream targeted genes Nanog [21] and FGF-BP1 [14] are two critical transcription factors that are considered to be able to regulate stem cell proliferation and self-renewal. Our study found that DCC-2036 could dramatically reduce the protein expression of KLF5 and its downstream targeted genes Nanog and FGF-BP1 in TNBC cell lines (Fig. 2A). More importantly, KLF5 was proved to be able to partially rescue the reduction of TNBC CSC mammospheres which was induced by DCC-2036 (Fig. 2B–D). To investigate the mechanism by which DCC-2036 reduced KLF5 expression in TNBC cell lines, we quantified the mRNA level of *KLF5* using quantitative RT-PCR. Although DCC-2036 significantly reduced the mRNA level of *Nanog*, which is a *KLF5* targeted gene, DCC-2036 did not reduce the mRNA level of *KLF5* in MDA-MB-231 cells (Fig. 2E). Hence, we estimated the effect of DCC-2036 on KLF5 protein stability. We analyzed the half-life of the KLF5 protein after treating with cycloheximide in MDA-MB-231 and HS-578T cell lines. Decay of KLF5 protein was remarkably accelerated by the addition of DCC-2036 (5 μM) (Fig. 2F). Furthermore, MG132, a proteasome inhibitor, effectively blocked the DCC-2036-induced decrease in KLF5 protein levels in the two cell lines (Fig. 2G), Additionally, we assessed the effect of DCC-2036 on KLF5 ubiquitin modifications directly, and indeed the ubiquitin modifications of KLF5 were also increased after DCC-2036 treatment (Fig. 2H). These data indicated that DCC-2036 could reduce the stability of KLF5 protein through the ubiquitin-proteasome pathway.

### DCC-2036 downregulates the expression of KLF5 by inhibiting AXL

**Fig. 3:**
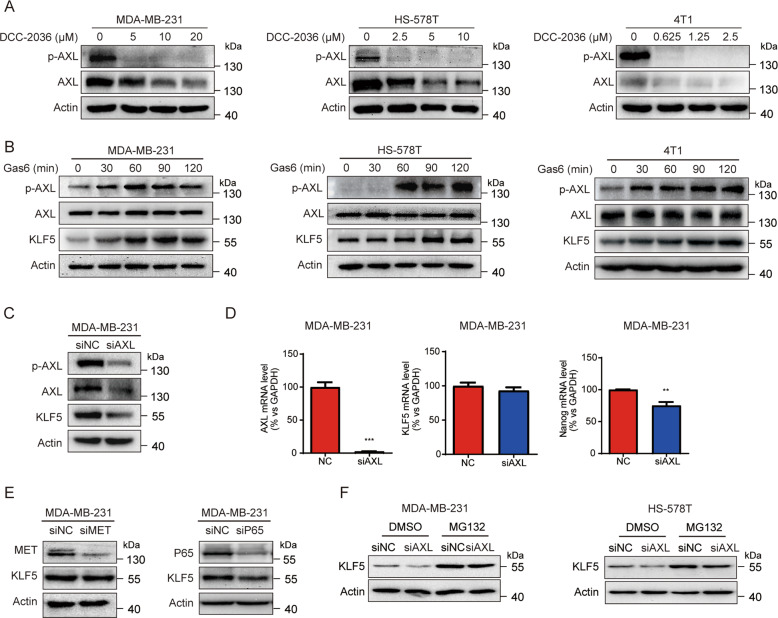
DCC-2036 decreases the KLF5 expression through the inhibition of AXL. **A** DCC-2036 inhibited the activation and expression of AXL in a dose-dependent manner. MDA-MB-231 cells, HS-578T, and 4T1 cells were treated with indicated concentrations of DCC-2036 for 48 h, and then the phosphorated and total levels of AXL were detected by WB. **B** Gas6 increased the protein levels of p-AXL and KLF5. MDA-MB-231 cells, HS-578T, and 4T1 cells were treated with Gas6 (200 ng/ml) for 0, 30, 60, 90, 120 min. **C** The knockdown of AXL reduced the protein levels of p-AXL, AXL, and KLF5. MDA-MB-231 cells were transfected with human AXL siRNA or control siRNA (NC) for 48 h, and then the protein levels were tested by WB. **D** The mRNA levels of *AXL*, *KLF5*, and *Nanog* were quantified by q RT-PCR. The statistical significance was determined by Student’s *t*-test, **P < 0.01. **E** The knockdown of both MET and P65 had no downregulation effect on KLF5. MDA-MB-231 cells were transfected with human MET siRNA/NC or P65 siRNA/NC for 48 h and then for WB assay. **F** MG132 reversed the decrease of KLF5 induced by AXL knockdown in MDA-MB-231 cells and HS-578T cells. The cells were transfected with AXL siRNA for 44 h and then were treated with MG132 (20 μM) for 4 h.

Preliminary experiments of our group have proved that DCC-2036 mainly exerts its anti-TNBC effect by targeting tyrosine kinase AXL [12]. To further explore whether the reduced stability of KLF5 induced by DCC-2036 is associated with AXL in TNBC cells, we conducted Western Blot and found that DCC-2036 also inhibited both AXL activity and expression in TNBC cells with the same concentration used to test KLF5 (Fig. 3A). Consistently, the AXL agonist, Gas6 (growth arrest-specific 6), significantly increased the protein levels of p-AXL and KLF5 (Fig. 3B). To further confirm the hypothesis that AXL positively regulated the stability of KLF5 protein, we used small interfering RNA (siRNA) to knockdown *AXL*. We observed that KLF5 protein expression decreased after knocking down *AXL* in MDA-MB-231 cells (Fig. 3C). However, the mRNA level of *KLF5* did not change significantly (Fig. 3F), but the mRNA level of *Nanog* was decreased (Fig. 3D). Considering that DCC-2036 can also target the tyrosine kinase MET and regulate the downstream PI3K/Akt-NF-κB signaling pathway in TNBC [12], we knocked down MET or P65 in MDA-MB-231 cells to detect the change of KLF5 expression (Fig. 3E). Results indicated that the protein level of KLF5 did not change, which further revealed that the decrease of KLF5 was mainly caused by the downregulation of *AXL*. In addition, MG132 also blocked the decrease of KLF5, which is induced by *AXL* knockdown (Fig. 3F). Thus, DCC-2036 could reduce the protein stability of KLF5 in TNBC cells by inhibiting AXL.

### DCC-2036 and AXL regulate KLF5 through Akt/GSK3β pathway

**Fig. 4:**
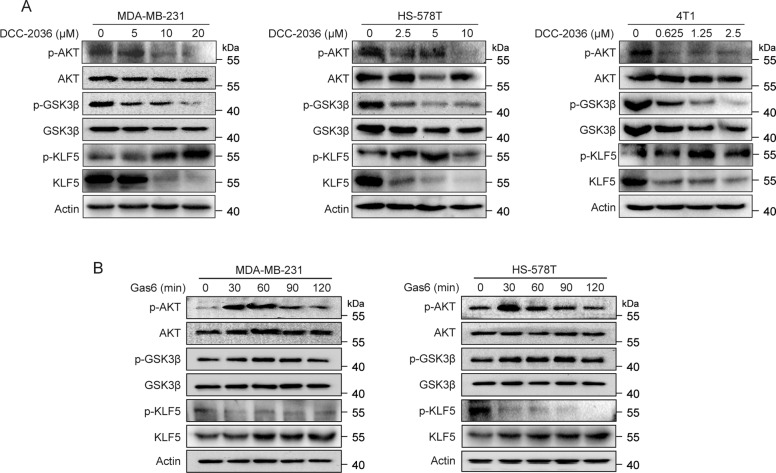
DCC-2036 and AXL regulate the KLF5 protein level through Akt/GSK3β pathway. **A** DCC-2036 decreased the phosphorylation of AKT (Ser473), GSK3β (Ser9), and total KLF5, and increased the phosphorylation of KLF5 (Ser303). TNBC cells were treated with DCC-2036 for 48 h, followed by the WB assay. **B** Gas6 promoted the increase of p-AKT, p-GSK3β, and total KLF5, as well as the decrease of p-KLF5 in MDA-MB-231 cells and HS-578T cells. Cells were treated with Gas6 (200 ng/ml) for 0, 30, 60, 90, and 120 min, and then examined by WB.

It has been reported that AXL can activate Akt, which then phosphorylates the S9 site of GSK3β and leads to the inactivation of GSK3β [22]. In addition, GSK3β can directly phosphorylate KLF5 (S303), which is ubiquitinated by SCF^Fbw7E3^ ubiquitin ligase and further degraded by 26 S proteasome [23, 24]. To determine whether Akt/GSK3β pathway mediated the degradation of KLF5 protein induced by DCC-2036 in TNBC cells, we examined the p-Akt (S473), p-GSK3β (S9), and p-KLF5 (S303) levels as well as the total KLF5 level after DCC-2036 treatment in MDA-MB-231, HS-578T and 4T1 cells. Data demonstrated that DCC-2036 reduced the level of p-Akt, p-GSK3β and total KLF5 as well as increased the level of p-KLF5 (Fig. 4A). In turn, the AXL agonist Gas6 increased the level of p-Akt, p-GSK3β and total KLF5 as well as decreased the level of p-KLF5 in MDA-MB-231 and HS-578T cells (Fig. 4B). Therefore, AXL may regulate the protein stability of KLF5 through the Akt/GSK3β signaling pathway and eventually mediate the inhibitory effect of DCC-2036 on TNBC stem cells.

### KLF5 upregulates AXL expression via directly binding to the AXL promoter in TNBC cells

**Fig. 5:**
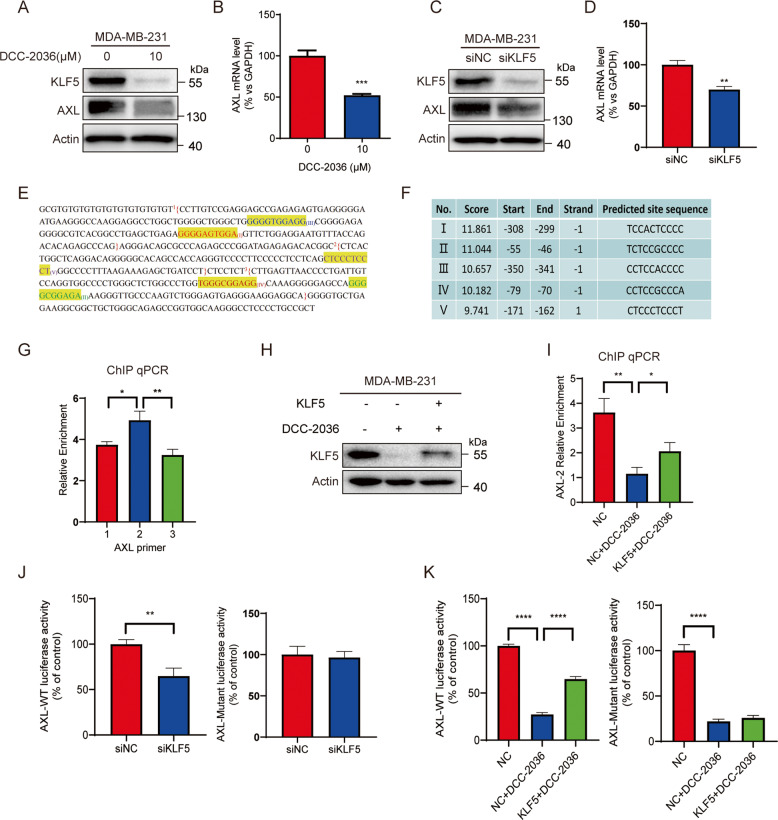
KLF5 upregulates AXL expression via binding to the promoter of *AXL*. **A**, **B** DCC-2036 downregulated the protein level and mRNA level of *AXL*. After the MDA-MB-231 cells were treated with DCC-2036 at 10 μM for 48 h, the protein and mRNA levels of *AXL* were measured by Western Blotting and qPCR. **C**, **D** Knockdown of *KLF5* decreased the protein level and mRNA level of *AXL*. MDA-MB-231 cells were transfected with *siKLF5* or control siRNA (siNC) for 48 h, followed by WB and qPCR. **E** Schematic representation of *AXL* promoter. I–V represented the putative site in the *AXL* promoter predicted by the Jasper database. One to three represented the qPCR products (fragments) by different *AXL* primers. **F** The information of the five highest-scoring putative sites (I–V). **G** Chromatin immunoprecipitation (ChIP) assay indicated that KLF5 is directly bound to *AXL* promoter mainly at fragment 2 in MDA-MB-231 cells. The results were shown as relative enrichment compared with IgG. **H**, **I** overexpression of KLF5 in MDA-MB-231 cells partially but significantly rescued the DCC-2036-induced reduction of KLF5 binding to fragment 2. MDA-MB-231 cells were transfected with KLF5 or vector control plasmid, followed by treating with 10 μM DCC-2036 for 48 h before WB and ChIP qPCR assay. **J** Regulation of *AXL* promoter activity by KLF5 in MDA-MB-231 cells. Wild-type (−2000 to +50 bp) or mutant promoter (site V was deleted) was cotransfected with siNC or *siKLF5*, and luciferase activity was measured with pRL-TK as an internal control. Data represent the mean of three independent experiments ± s.d. **K** Regulation of *AXL* promoter activity by DCC-2036 and overexpression of KLF5 in MDA-MB-231 cells. Wild-type or mutant promoter was cotransfected with KLF5 or vector control plasmid, followed by treating with 10 μM DCC-2036 for 48 h, and then luciferase activity was measured.

As a tyrosine kinase inhibitor, DCC-2036 mainly inhibits the activity (phosphorylation) of AXL. However, we found that the protein expression level and mRNA level of *AXL* have also been significantly downregulated (Fig. 5A, B). KLF5 was also decreased remarkably by DCC-2036 (Fig. 5A), so we speculated that KLF5 might positively regulate the expression of AXL at the transcriptional level. As shown in Fig. 5C, D, silencing KLF5 indeed downregulated the protein and mRNA levels of *AXL* in MDA-MB-231 cells. We further explored whether KLF5 could directly bind to the promoter of *AXL*. By analyzing the promoter sequence (−2000 to +50 bp) of *AXL* using the Jasper database, we found the five highest-scoring putative sites (I–V, Fig. 5E, F). To identify which site is the most important one, we first designed three primers for the ChIP qPCR assay to divide the promoter into three fragments. Fragment 1 (−418 to −237 bp) included sites III and I, fragment 2 (−266 to −136 bp) included site V, and fragment 3 (−128 to −9 bp) included site IV and II (Fig. 5E). The results demonstrated that KLF5 could directly bind to *AXL* promoter in all the three fragments and the fragment 2 has the highest degree of enrichment, which indicated that KLF5 mainly bound to fragment 2 (Fig. 5G). The rescue assay of ChIP qPCR with *AXL*-2 Primer demonstrated that the binding ability of KLF5 on fragment 2 was lowered by DCC-2036, but could be significantly reversed by the overexpression of KLF5 (Fig. 5H-I). To rule out nonspecific binding, we designed a new pair of AXL primers which could bind to −2702 to −2685 bp and −2550 to −2531 bp of the AXL gene, and the PCR product is a KLF5 unrelated and distant region in the AXL gene. As we expected, the CHIP qPCR assay indicated a negative result. The melting curve of the AXL-CHIP primer showed obvious double peaks (Fig. S1A), and the primers were tested as specific primers (Fig. S1B), indicating that the sample could not obtain the fragment amplified by the new primers after CHIP. Therefore, the KLF5 protein could not bind to this unrelated fragment (−2702 to −2531 bp) (Fig. S1). To further verify that KLF5 mainly binds to site V in fragment 2, the *AXL*-WT promoter (−2000 to+50 bp) and *AXL*-Mutant promoter (the site V was deleted from −2000 to+50 bp) were cloned, and the luciferase assay was performed. As shown in Fig. 5J, knockdown of KLF5 could reduce the luciferase activity of the *AXL*-WT promoter but not the *AXL*-mutant promoter, indicating that site V (−171 to −162 bp) was the critical KLF5 binding site. In addition, overexpression of KLF5 in MDA-MB-231 cells partially but significantly rescued the DCC-2036-induced reduction of luciferase activity of the *AXL*-WT promoter. Although DCC-2036 also reduced the luciferase activity of the *AXL*-Mutant promoter, KLF5 overexpression could not reverse this change (Fig. 5K).

### DCC-2036 inhibits CSCs in vivo

**Fig. 6:**
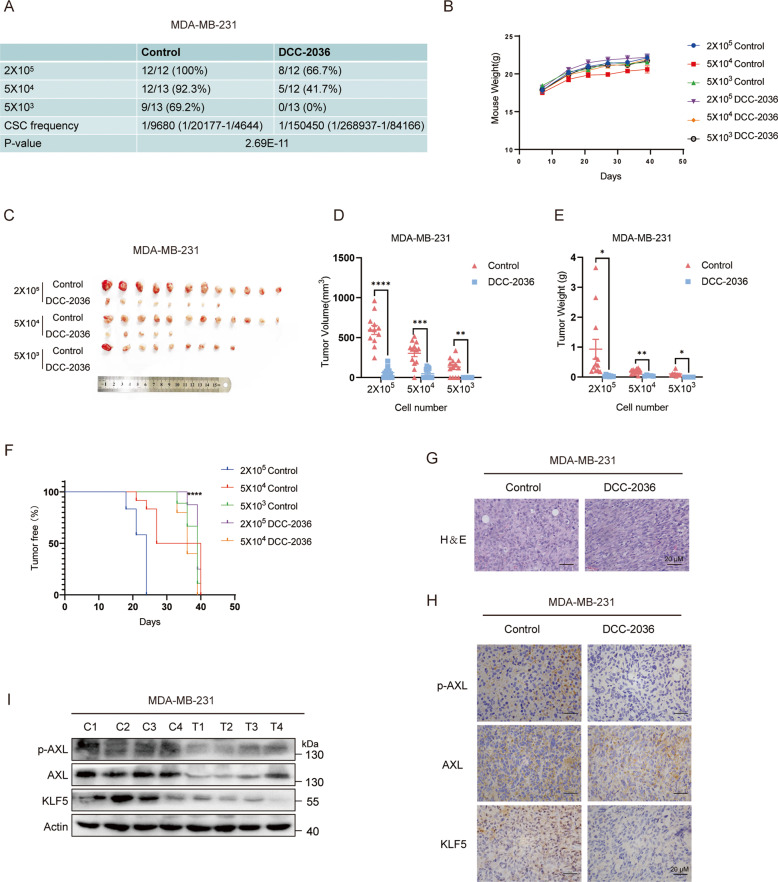
DCC-2036 inhibits CSCs in vivo according to the limited dilution assay by pretreating MDA-MB-231 cells with DCC-2036 or DMSO for 48 h. **A** DCC-2036 inhibited tumorigenicity of CSCs in vivo on the basis of the limited dilution assay. MDA-MB-231 cells, treated with DCC-2036 (5 μM) or DMSO for 48 h, were transplanted into the mammary fat pad of BALB/C-nude mice in limiting dilutions respectively: 200,000 (*n* = 12 and 12), 50,000 (*n* = 13 and 12), or 5000 (*n* = 13 and 13) cells per injection site. Tumorigenesis was recorded after 1 week. The frequency of breast cancer stem cells (BCSCs) was calculated by ELDA (extreme limiting dilution analysis, http://bioinf.wehi.edu.au/software/elda/). A statistically significant difference was detected between groups. **B** The body weight variation of BALB/C-nude mice over the course of the experiment. **C** Images of collected tumor xenografts from nude mice at the termination of the experiment. **D**, **E** Comparisons of xenograft tumor size and weight between groups at the end of experiments. The statistical significance was determined by Student’s *t*-test. **F** The curves showed the probability of tumor-free survival vs days in the nude mice injected with different numbers of MDA-MB-231/control and MDA-MB-231/DCC-2036 cells. The statistical significance was determined by a log-rank test. ****P < 0.0001. **G** The effect of DCC-2036 on the xenograft tissues in BALB/C-nude mice. Hematoxylin and eosin (H&E) staining indicates the histology of tumor tissues. Scale bars, 20 μm. **H** Immunohistochemical analysis using p-AXL, AXL, and KLF5 antibodies in xenograft tissues from BALB/C-nude mice. Scale bars, 20 μm. **I** Immunoblot of xenograft tissues from BALB/C-nude mice posterior to the experiments. C means Control, T means DCC-2036. We chose the tumor tissues randomly for H&E staining, immunohistochemical analysis, and immunoblotting.

**Fig. 7:**
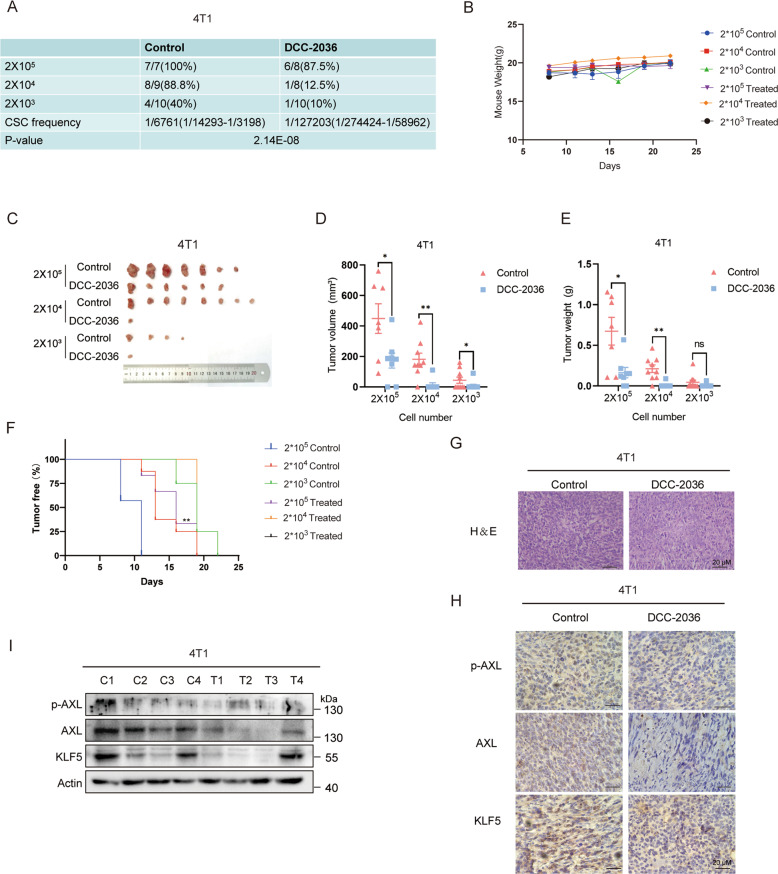
DCC-2036 inhibits CSCs in vivo according to the limited dilution assay by pretreating 4T1 cells with DCC-2036 or DMSO for 48 h. **A** In vivo tumorigenicity assay with limited dilution using DMSO or DCC-2036 treated 4T1 cells respectively: 200,000 (*n* = 7 and 8), 20,000 (*n* = 9 and 8), or 2000 (*n* = 10 and 10) cells per injection site. The frequency of BCSCs was calculated by ELDA. **B** Weight variation of mice following transplant of 4T1 cells into BALB/C mice after 7 days. **C** Images of tumor formation in BALB/C mice. **D**, **E** Transplanted tumors were harvested, and the tumor size and weight were measured at the end of the experiment. The statistical significance was determined by Student’s *t*-test. **F** Tumor-free survival curve of BALB/C mice is shown. **G** Hematoxylin and eosin (H&E) staining indicates the histology of tumor tissues. Scale bars, 20 μm. **H** Immunohistochemical analysis using p-AXL, AXL, and KLF5 antibodies in xenograft tissues from BALB/C mice. Scale bars, 20 μm. **I** Immunoblot of transplanted tumors from BALB/C mice posterior to the experiments. C means Control, T means DCC-2036. We chose the tumor tissues randomly for H&E staining, immunohistochemical analysis, and immunoblotting.

The limited dilution assay in vivo is crucial for defining the phenotype of CSC [25]. Thus, we pretreated MDA-MB-231 cells and 4T1 cells with DCC-2036 or DMSO for 48 h, followed by serial dilutions of MDA-MB-231 cells and 4T1 cells before injection to evaluate their tumorigenicity in vivo. As shown in Fig. 6A, C, MDA-MB-231 cells implanted at a cell density of 2 × 10^5^ formed tumors in 100% of the control group, whereas the tumor formation rate was decreased to 66.7% in the DCC-2036-treated group. When a number of 5 × 10^4^ MDA-MB-231 cells were implanted, the tumor formation rate was 92.3% in the control group and the rate in the DCC-2036-treated group plummeted to 41.7%. Remarkably, implantation with 5000 cells still formed tumors with 69.2% efficiency in the control group, whereas no tumor formation in the DCC-2036-treated group was observed (Fig. 6C). Furthermore, DCC-2036 treatment prominently decreased the tumor volumes and weights (Fig. 6D, E), whereas mice body weights showed no obvious difference between control and DCC-2036 treatment group (Fig. 6B). Notably, DCC-2036 treatment group dramatically prolonged the tumor-free survival of BALB/C-nude mice (2 × 10^5^ group) (Fig. 6F). Immunohistochemical analysis revealed that the expression of p-AXL, AXL and KLF5 was markedly downregulated by DCC-2036 treatment in BALB/C-nude mice (Fig. 6G, H). Immunoblotting of transplanted tumor tissues from BALB/C-nude mice indicated that DCC-2036 significantly inhibited p-AXL, AXL, and KLF5 (Fig. 6I). Similar results were also observed when 4T1 cells were utilized for serial dilutions (Fig. 7A–C). DCC-2036 treatment obviously decreased tumor weights (Fig. 7D) and volumes (Fig. 7E) of 4T1 tumor-bearing mice and exhibited longer tumor-free survival (Figs. 7F, 2 × 10^5^ group). Moreover, compared with the control group, Immunohistochemical analysis and Immunoblotting showed that DCC-2036 dramatically attenuates p-AXL, AXL, and KLF5 expression (Fig. 7G–I). Those results indicated that DCC-2036 showed a suppressive effect on CSCs in vivo.

### The expression of p-AXL and AXL positively correlates with the expression of KLF5 in human TNBC specimens respectively

**Fig. 8:**
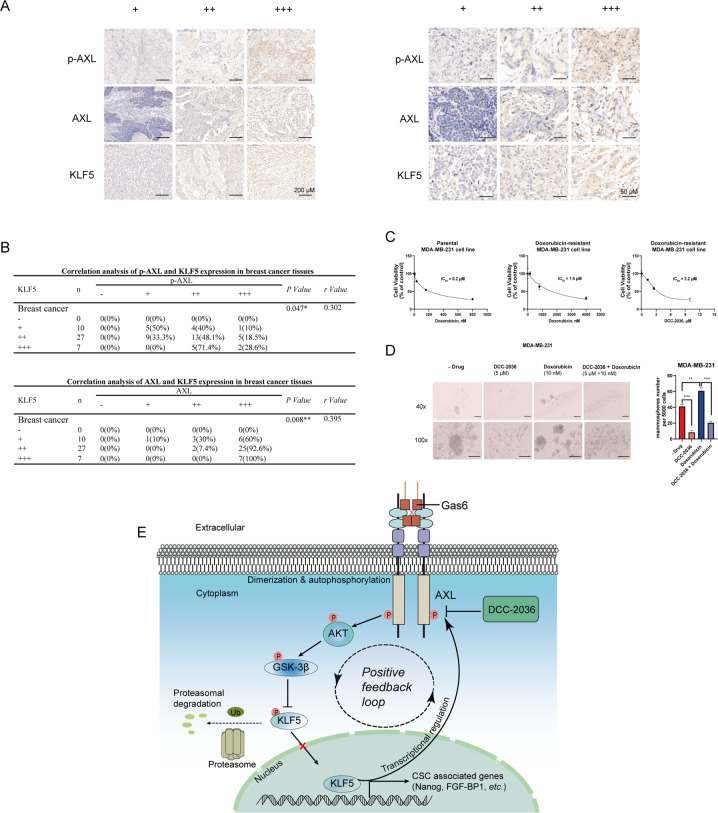
The expression of p-AXL and AXL positively correlates with the expression of KLF5 in human TNBC specimens. Moreover, DCC-2036 increases the sensitivity of TNBC chemotherapy by decreasing BCSCs. **A** Representative immunohistochemical staining images of p-AXL, AXL, and KLF5 protein in TNBC specimens, in which the expression of p-AXL, AXL, and KLF5 proteins was indicated by mild positive (+), moderate positive (++), and strong positive (+++), respectively. Left: Scale bars, 200 μm (magnification 40×); Right: Scale bars, 50 μm (magnification 100×). **B** The expression levels of p-AXL, AXL, and KLF5 in TNBC samples were positively correlated. The statistical significance was determined by Spearman correlation analysis. R-value is the correlation coefficient. *P < 0.05, **P < 0.01. **C** The cell viability analysis of MDA-MB-231 and the doxorubicin-resistant MDA-MB-231 cells after treating with different concentrations of doxorubicin and DCC-2036 (*n* = 3). **D** The number and size of mammospheres formed following treatment with doxorubicin (10 nM), DCC-2036 (5 μM), and a combination of doxorubicin and DCC-2036 in MDA-MB-231 cells. Scale bars, 100 μm (magnification 40×); Scale bars, 100 μm (magnification 100×). The histogram represents the mean value of three experiments. The statistical significance was determined by Student’s *t*-test. **P < 0.01, ***P < 0.001. **E** Schematic representations of the inhibitory effect of DCC-2036 on triple-negative breast cancer stem cells through AXL-KLF5 positive feedback loop. In the progression of TNBC, AXL can activate Akt, which subsequently increases GSK3β (S9) phosphorylation and leads to the GSK3β inactivation. However, GSK3β-mediated KLF5 (S303) phosphorylation level promotes the KLF5 ubiquitination by SCF^Fbw7E3^ ubiquitin ligase and degradation by 26 S proteasome. Thus, KLF5 into the nucleus promotes the expressions of stemness-associated transcription factors, and in turn, KLF5 binds to the *AXL* promoter region (−171 to −162 bp), which forms a positive feedback regulation mechanism further to facilitate tumorigenesis. DCC-2036 inhibits the expression of KLF5 via the AXL-Akt-GSK3β signal axis and further disrupts the AXL-KLF5 positive feedback loop. Ultimately, DCC-2036 inhibits TNBC stem cells and suppresses TNBC development.

Since activation of AXL led to KLF5 stabilization, which in turn upregulated AXL at the transcriptional level, the positive feedback regulation mechanism of AXL-KLF5 might mediate the inhibitory effect of DCC-2036 on TNBC stem cells. To further verify the existence of this positive feedback loop, we detected the correlation between the expression of AXL and KLF5 proteins. Immunohistochemical staining was conducted on 44 paraffin tissue samples of triple-negative breast cancer. Significantly positive correlations between p-AXL and KLF5 (*r* = 0.302, *P* = 0.047) as well as AXL and KLF5 (*r* = 0.395, *P* = 0.008) were detected in these TNBC samples (Fig. 8A, B). Strikingly, p-AXL, AXL, and KLF5 positivity was observed in 100% (44 of 44) of TNBC samples, which suggested that p-AXL, AXL, and KLF5 were greatly enriched in human TNBC (Fig. 8B). These results further confirmed the existence of the AXL-KLF5 positive feedback loop in TNBC, and it could be considered as the potential therapeutic target for TNBC treatment.

### DCC-2036 increases the sensitivity of TNBC chemotherapy by decreasing BCSCs

The failure to achieve long-term survival of TNBC patients is greatly due to tumor relapse caused by chemotherapy-resistant breast cancer stem cells [26]. We constructed a doxorubicin-resistant MDA-MB-231 cell line and showed by MTS assay that the IC50 value of doxorubicin increased from 0.2 μM to 1.6 μM, demonstrating that the doxorubicin-resistant cell line has been successfully established. Then, we found that DCC-2036 showed potent inhibitory ability in doxorubicin-resistant cells (IC50 = 3.2 μM), which is similar to that in parental cells (IC50 = 3.3 μM)[12] (Fig. 8C). This experiment indicates that DCC-2036 can overcome the resistance of TNBC chemotherapy in some extent. To confirm that these effects were caused by BCSCs, we evaluated the stemness characteristics followed by treating with doxorubicin and/or DCC-2036 using a mammospheres formation assay. These results suggested that chemotherapy (doxorubicin) enhanced the mammospheres formed capacity, which meant the enrichment of BCSCs, but this enrichment was significantly decreased after DCC-2036 treatment (Fig. 8D). Therefore, it implied that DCC-2036 increased the sensitivity of TNBC chemotherapy by decreasing BCSCs.

In conclusion, our study indicated that DCC-2036 significantly represses TNBC stem cells by targeting the AXL-KLF5 positive feedback loop (Fig. 8E).

## Discussion

Triple-negative breast cancer (TNBC) is generally defined by the lack of the expression of estrogen receptor (ER), progesterone receptor (PR), and HER2 [27]. TNBC is an aggressive type of breast cancer, which is prone to recurrence and metastasis, leading to a low survival rate [28]. Breast cancer stem cells (BCSCs) comprise only a minority of populations within breast cancer cells and play a prominent role in cancer relapse, metastasis, and chemoresistance [29–31]. At present, the strategies for effectively eradicating or inhibiting TNBC cancer stem cells (CSCs) are still limited, so the study of novel drugs with anti-CSCs function is of great value for the treatment of TNBC. Recently, aberrant tyrosine kinase (TK) signaling mechanisms have attracted extensive attention in cancer stem cell biology [32]. Small-molecule tyrosine kinase inhibitors (TKIs) have been successfully developed and extensively utilized in clinical practice [12, 33, 34], including imatinib, erlotinib, gefitinib, and so on. DCC-2036 is a novel third-generation TKI, which has a broad kinase inhibition spectrum, including ABL, FLT3, TIE2, SRC, PDGFRα, FYN, AXL, and MET [11]. We previously reported that DCC-2036 could significantly inhibit the proliferation of TNBC cells, and its inhibitory effect was better than that of cisplatin, gemcitabine, and other commonly used clinical drugs. Besides, DCC-2036 could induce apoptosis, inhibit migration, and invasion of several TNBC cell lines in vitro. More importantly, DCC-2036 showed pronounced antitumor activity in the xenograft NSG model and the TNBC PDX model without significant toxicity [12]. Moreover, the critical target of DCC-2036 in TNBC cells was AXL [12]. Studies have shown that AXL was highly expressed in TNBC cells [35]. AXL could induce EMT and participate in the regulation of BCSCs function [22]. However, the underlying mechanisms still need further investigation. Thus, we speculated that DCC-2036 might also inhibit TNBC stem cells, and our study was designed to investigate the possible suppressive effect of DCC-2036 on TNBC stem cells and its mechanism.

Overall, our study demonstrated that DCC-2036 significantly inhibited CSCs in vitro and in vivo (Figs. 1, 6, 7). It has been reported that KLF5 overexpression played a significant role in the self-renewal ability of cancer stem cells and was associated with stemness [36, 37]. Mifepristone and metformin inhibited CSCs by inhibiting KLF5 [15, 38]. Herein, the KLF5 suppression was also found to be responsible for the inhibitory effect of DCC-2036 on CSCs (Fig. 2). Moreover, it was revealed that the downregulation of KLF5 was mediated by the ubiquitin-proteasome pathway (Fig. 2). Our research found that DCC-2036 downregulated the expression of KLF5 by inhibiting the activity of AXL (Fig. 3). Besides, DCC-2036 and AXL regulated KLF5 protein through Akt/GSK3β pathway. The phosphorylation of KLF5 enhanced the protein ubiquitination by SCF^Fbw7^, facilitating proteasomal degradation of KLF5. (Fig. 4). Interestingly, we found that DCC-2036 not only reduced the phosphorylated level of AXL, but also the total protein level and mRNA level of *AXL* (Fig. 5). To explore whether KLF5, which was a transcriptional factor downstream of AXL, could, in turn, increase the expression of AXL to form an AXL-KLF5 positive feedback loop, we first used CHIP qPCR to test the binding ability of KLF5 and different *AXL* Promoter fragments according to the predicted sites by Jasper database. Results revealed that KLF5 bound to fragment 2 (−266 to −136 bp) included site V (−171 to −162 bp) most (Fig. 5G). Furthermore, overexpression of KLF5 in MDA-MB-231 cells partially but significantly rescued the DCC-2036-induced reduction of KLF5 binding to fragment 2 (Fig. 5I). Additionally, silencing *KLF5* could reduce the luciferase activity of *AXL*-WT promoter (−2000 to +50 bp) but not *AXL*-Mutant promoter (the site V was deleted from −2000 to +50 bp) (Fig. 5J), indicating that the site V (−171 to −162 bp) was the critical KLF5 binding site. Overexpression of KLF5 in MDA-MB-231 cells could rescue DCC-2036-induced reduction of luciferase activity of AXL-WT promoter but not AXL-Mutant promoter (Fig. 5K), which revealed the key role of site V (−171 to −162 bp) in AXL downregulation by DCC-2036. However, DCC-2036 also reduced the luciferase activity of the AXL-Mutant promoter (Fig. 5K, right). It aroused our attention. In addition to KLF5, AXL is an EMT-induced regulator of cancer metastasis, controlled by Slug and Vimentin in breast cancer [17, 39–41]. Thus, the total AXL protein levels might also be lowered by EMT transcription factors, such as Slug or other transcription factors that have been changed by DCC-2036, which should be further investigated. Thereby, a decline of KLF5 by DCC-2036 was the critical but not the only factor for AXL downregulation, which was consistent with Fig. 5I. Data obtained in the 4T1 model in vivo seem less pronounced compared to the MDA-MB-231 model (Figs. 6, 7), especially with regard to the nuclear localization of KLF5, which is not well defined in 4T1 tumors. Some studies on transcriptomic profiling of different breast cancer mouse models showed that 4T1, despite triple-negative from the point of view of the hormone receptors, shows an arrangement more similar to luminal tumors [42] when evaluated from the transcriptomic point of view. In reality, we have found that DCC-2036 was more sensitive in most TNBC cell lines [12]. Therefore, the specificity of DCC-2036 on TNBC might be related to the different KLF5 staining. In TNBC specimens, the expression levels of p-AXL/AXL was positively correlated with KLF5 expression (Fig. 8). These results further confirmed the existence of the AXL-KLF5 positive feedback loop. Hence, we highlighted that targeting the AXL-KLF5 positive feedback loop could suppress TNBC CSCs, and thus enhance the efficacy of antitumor therapy (Fig. 8).

In summary, the present study provides evidences for the suppressive function of DCC-2036 on TNBC CSCs in vitro and in vivo. The existence of positive feedback regulation between AXL and KLF5 was revealed in TNBC, which could be blocked by DCC-2036. Our study highlights the potential clinical value of DCC-2036 for the treatment of TNBC with the overexpression of AXL and KLF5 via the repression of CSCs.

## Supplementary information

supplementary Figure legend

Figure 1S

aj-checklist

## Data Availability

The data that support the findings of this study are available from the corresponding author upon request.
