# Supplementary material for: Inhibitory effect of the novel tyrosine kinase inhibitor DCC-2036 on triple-negative breast cancer stem cells through AXL-KLF5 positive feedback loop
Source: Cell Death Dis. 2022 Aug 30;13(8):749. doi: 10.1038/s41419-022-05185-x (PMC9428169; doi:10.1038/s41419-022-05185-x)
Supplement: Supplementary file 1 — supplementary Figure legend [file 41419_2022_5185_MOESM1_ESM.docx]

**Fig. S1: The KLF5 protein could not bind to the unrelated fragment (-2702bp ~ -2531bp) since the sample could not obtain the fragment amplified by the new primers after CHIP.**

A, The Amplification curve and Melting curve of the AXL-CHIP primer for KLF5 binding. B, The Amplification curve and Melting curve for primer test. C, The figure of DNA X-CHIP gel. KB, control; NC, vector control plasmid; NC+DCC, vector control plasmid+DCC-2036; KLF5+DCC, KLF5 plasmid+DCC-2036. MDA-MB-231 cells were transfected with KLF5 or vector control plasmid, followed by treating with 10 μM DCC-2036 for 48 h before ChIP assay.
