# Supplementary figures and images for: Inhibitory effect of the novel tyrosine kinase inhibitor DCC-2036 on triple-negative breast cancer stem cells through AXL-KLF5 positive feedback loop
Source: Cell Death Dis. 2022 Aug 30;13(8):749. doi: 10.1038/s41419-022-05185-x (PMC9428169; doi:10.1038/s41419-022-05185-x)

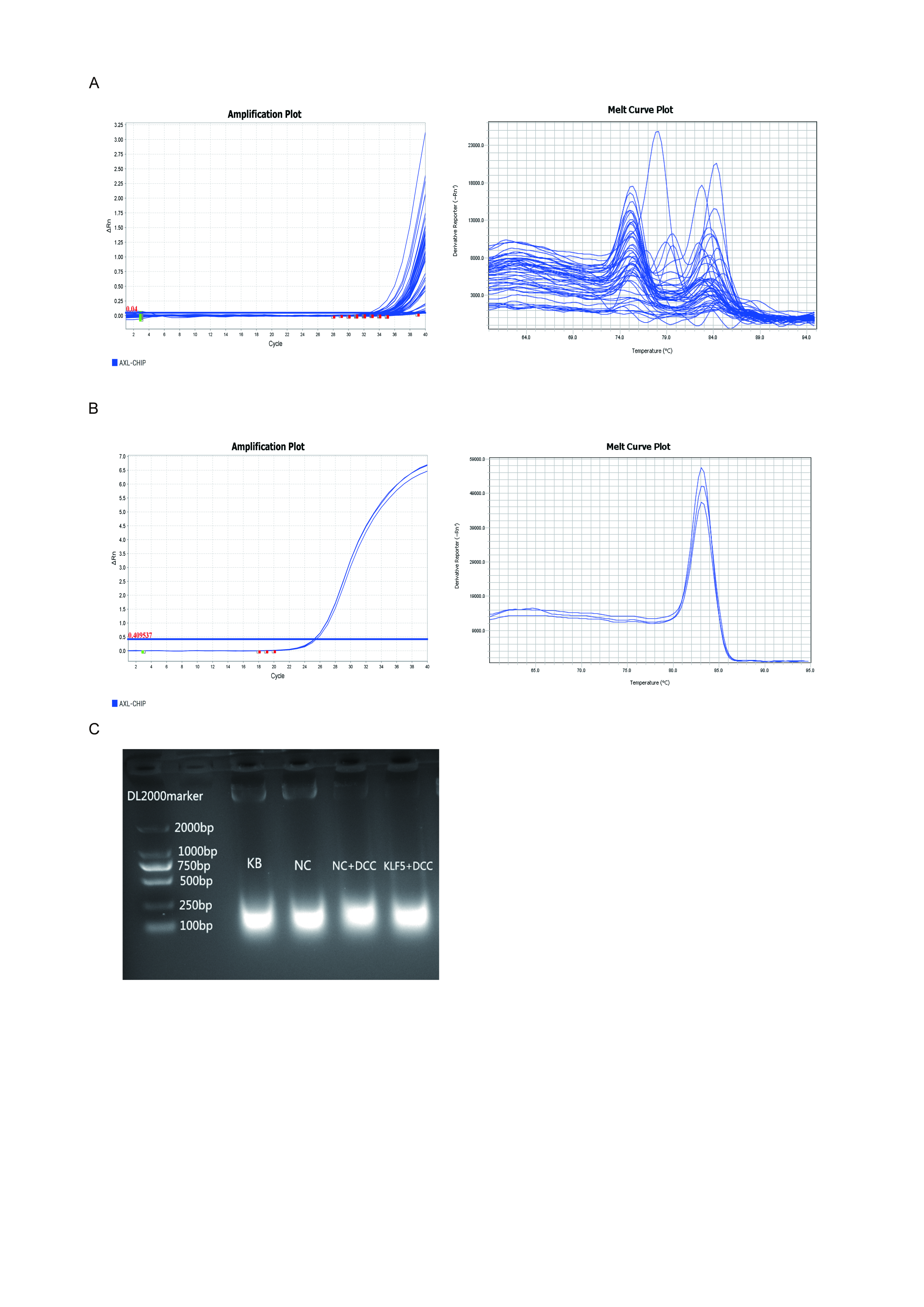

Supplement: Supplementary file 2 — Figure 1S [file 41419_2022_5185_MOESM2_ESM.tif]
